# Draft Genome Sequences of the Black Truffles Tuber brumale Vittad. and Tuber indicum Cook & Massee

**DOI:** 10.1128/MRA.00799-20

**Published:** 2021-01-28

**Authors:** Emmanuelle Morin, Claude Murat, Nicolas Cichocki, Herminia De la Varga, Annegret Kohler, Jianping Xu, Igor V. Grigoriev, Francis M. Martin

**Affiliations:** a Université de Lorraine, INRAE, IAM, Nancy, France; b McMaster University, Department of Biology, Hamilton, Canada; c DOE Joint Genome Institute, Lawrence Berkeley National Laboratory, Berkeley, California, USA; Vanderbilt University

## Abstract

Tuber brumale and Tuber indicum (Pezizomycetes) are two edible black truffles establishing ectomycorrhizal symbiosis with trees and shrubs. *T. brumale* is ubiquitous in Europe, and *T. indicum* is mainly found in China. Here, we present the draft genome sequences of *T. brumale* and *T. indicum*.

## ANNOUNCEMENT

The black truffles Tuber brumale Vittadini and Tuber indicum Cook & Massee are ectomycorrhizal ascomycetes. *T. brumale* is widespread in Europe, except in the boreal and Arctic regions (1). This species often competes with the Périgord black truffle (Tuber melanosporum) in truffle orchards (2). *T. indicum* is found mainly in the Chinese provinces of Yunnan and Sichuan (3). The two species belong to the Melanosporum phylogenetic clade (4) and have morphological features similar to those of *T. melanosporum*, making their distinctions sometimes difficult (5). Together with the published genome sequences of T. aestivum, T. borchii, T. magnatum, and *T. melanosporum* (6–8), these newly sequenced genomes will allow a better understanding of the evolution, biology, and ecology of truffles.

For genome and RNA sequencing, a *T. brumale* fruiting body was harvested in Lozère (Occitanie, France) in March 2014, and a *T. indicum* fruiting body was purchased at a French market in 2013. For both species, genomic DNA (gDNA) was extracted from 2 g of fruiting body by using a modified cetyl trimethylammonium bromide (CTAB) protocol (9). Total RNA was extracted using the RNeasy plant minikit (Qiagen) as described earlier (6). The gDNA and the Illumina TruSeq Nano kit were used to construct paired-end libraries (2 × 100 bp for both and 2 × 125 bp for *T. brumale*) as well as mate pair libraries (with insert sizes of 3 and 8 kbp) using the Illumina Nextera mate pair kit. In addition, paired-end libraries (2 × 100 bp and 2 × 125 bp) were generated from total RNA using the Illumina TruSeq stranded mRNA kit. Sequencing was performed at the GeT-PlaGe sequencing facility (Toulouse, France) using the Illumina HiSeq 2500 platform. The raw Illumina reads were trimmed of adapter sequences and low-quality bases using Trimmomatic v.0.32 (10) with the following parameters: TRAILING:20, LEADING:20, SLIDINGWINDOW:4:20, and MINLEN:70. Assembly of the genomes was carried out using ALLPATHS-LG v.46154 (11) and GapCloser v.1.12.6 (12). The genome assemblies were then annotated using the Joint Genome Institute (JGI) annotation pipeline (13, 14).

The sequencing data statistics are shown in Table 1. The genome sizes of *T. brumale* and *T. indicum* are in the range of other truffle species, from 97.18 to 192 Mb (6–8).

**TABLE 1:** Genomic features and raw data of *Tuber brumale* and *Tuber indicum*

Organism	Source	No. of reads	Draft genome size (Mb)	No. of scaffolds	*N*_50_ (bp)	G+C content (%)	Mean coverage (×)	SRA accession no.	GenBank accession no.	BioProject accession no.
*Tuber brumale*	DNA	136,348,163	171.44	1,475	336,267	46.46	131.63	SRR12018987, SRR12018988, SRR12018989	JACCEG000000000	PRJNA633036
		135,457,273						SRR12018993, SRR12018994,SRR12018995		
	RNA	128,865,953						SRR12018990, SRR12018991, SRR12018992		
										
*Tuber indicum*	DNA	558,521,206	110.49	734	538,733	47.41	239.48	SRR12104989, SRR12104990, SRR12104991	JACCEH000000000	PRJNA633038
	RNA	86,652,466						SRR12104986, SRR12104987, SRR12104988		

RepeatScout v.1.0.5 (15) was used to identify *de novo* repetitive DNA in the genome assemblies as reported by Peter et al. (16). RepeatMasker v.4.0.9 (17) was used to estimate the repeat element coverage in the genomes. Transposable elements constitute 61.5% and 47.1% of the *T. brumale* and *T. indicum* genomes, respectively. Default parameters were used for all software except where otherwise noted.

A total of 12,380 protein-coding genes for *T. brumale* and 11,870 protein-coding genes for *T. indicum* were predicted. The number of protein-coding genes is also in the range of other truffle species, from 9,344 to 12,346 protein-coding genes (6–8).

### Data availability.

The draft whole-genome shotgun projects were deposited in DDBJ/ENA/GenBank. The SRA and GenBank accession numbers for *T. brumale* and *T. indicum* are listed in Table 1. The genome assemblies and annotations are also available at the JGI-DOE Mycocosm portal (13) (https://mycocosm.jgi.doe.gov/Tubbr1_1 and https://mycocosm.jgi.doe.gov/Tubin1_1).
